# Cytotoxic mechanisms of pemetrexed and HDAC inhibition in non-small cell lung cancer cells involving ribonucleotides in DNA

**DOI:** 10.1038/s41598-025-86007-w

**Published:** 2025-01-15

**Authors:** Tobias Solli Iveland, Lars Hagen, Mirta Mittelstedt Leal de Sousa, Nina Beate Liabakk, Per Arne Aas, Animesh Sharma, Bodil Kavli, Geir Slupphaug

**Affiliations:** 1 Department of Clinical and Molecular Medicine, Norwegian University of Science and Technology, N-7491 Trondheim, Norway; 2 The Cancer Clinic, St. Olavs Hospital, Trondheim, Norway; 3 The Proteomics and Metabolomics Core Facility, PROMEC, at NTNU and the Central Norway Regional Health Authority, Trondheim, Norway; 4 Clinic of Laboratory Medicine, St. Olavs Hospital, N-7491 Trondheim, Norway; 5 Centre for Embryology and Healthy Development, University of Oslo, 0373 Oslo, Norway

**Keywords:** Non-small cell lung cancer (NSCLC), Pemetrexed, HDAC inhibitors, Uracil, UNG, Ribonucleotide misincorporation, Cancer, Molecular medicine

## Abstract

The cytotoxic mechanisms of thymidylate synthase inhibitors, such as the multitarget antifolate pemetrexed, are not yet fully understood. Emerging evidence indicates that combining pemetrexed with histone deacetylase inhibitors (HDACi) may enhance therapeutic efficacy in non-small cell lung cancer (NSCLC). To explore this further, A549 NSCLC cells were treated with various combinations of pemetrexed and the HDACi MS275 (Entinostat), and subsequently assessed for cell viability, cell cycle changes, and genotoxic markers. Proteomic alterations were analyzed using label-free shotgun and targeted LC–MS/MS. MS275 enhanced the sensitivity of A549 cells to pemetrexed, but only when administered following prior treatment with pemetrexed. Both HeLa (p53 negative) and A549 (p53 positive) showed robust activation of γH2AX upon treatment with this combination. Importantly, CRISPR/Cas9 knockout of the uracil-DNA glycosylase UNG did not affect γH2AX activation or sensitivity to pemetrexed. Proteomic analysis revealed that MS275 altered the expression of known pemetrexed targets, as well as several proteins involved in pyrimidine metabolism and DNA repair, which could potentiate pemetrexed cytotoxicity. Contrary to the conventional model of antifolate toxicity, which implicates futile cycles of uracil incorporation and excision in DNA, we propose that ribonucleotide incorporation in nuclear and mitochondrial DNA significantly contributes to the cytotoxicity of antifolates like pemetrexed, and likely also of fluorinated pyrimidine analogs. HDAC inhibition apparently exacerbates cytotoxicity of these agents by inhibiting error-free repair of misincorporated ribonucleotides in DNA. The potential of HDACis to modulate pyrimidine metabolism and DNA damage responses offers novel strategies for improving NSCLC outcomes.

## Introduction

Lung cancer remains the leading cause of cancer-related deaths worldwide, with non-small cell lung cancer (NSCLC) accounting for most cases. Pemetrexed (PMX), an antifolate agent, is central to NSCLC treatment, both as monotherapy and in combination with immunotherapy^1^. PMX primarily targets thymidylate synthase (TYMS), and its efficacy is inversely correlated with TYMS expression^2^. PMX also inhibits other enzymes in purine and pyrimidine biosynthesis, including DHFR, GART and ATIC, with increased DHFR and GART expression linked to PMX resistance^3,4^. Other DNA metabolism factors, such as RRM1, DUT and DNA repair proteins (UNG, ERCC1, CHEK1, MSH2, XRCC5) have also been associated with PMX resistance^5^.

Despite the long-standing use of antifolates and nucleotide analogs that inhibit TYMS, the cytotoxic mechanisms underlying their action remain inadequately understood. TYMS inhibition leads to reduced dTTP, disruption of DNA replication and induces “thymineless death” (TLD)^6^. Reduced dTTP leads to enhanced dUMP misincorporation, and the extent of misincorporation depends on the abundance of dUTPase (DUT). DUT knockdown enhances PMX sensitivity^7^, suggesting uracil misincorporation as a key cytotoxic mediator. Although misincorporated uracils (U:A) are not miscoding, removal by DNA glycosylases (UDGs) generates cytotoxic and mutagenic apyrimidinic (AP) sites^8^.

UNG is the dominant UDG in humans, with UNG1 (mitochondrial) and UNG2 (nuclear) isoforms^9^. Recent work from our group has also identified a distinct nuclear UNG1 variant that processes genomic uracil^10^. UNG2 harbors a PCNA-interacting motif at its extreme N-terminal, whereas both isoforms harbor RPA-binding motifs adjacent to the common catalytic domain. The PCNA-binding motif of UNG2 renders it especially suited for effective post-replicative removal of misincorporated uracil^11^. After uracil excision, the resulting AP sites are cleaved by an AP endonuclease, and DNA polymerases and ligases complete error-free repair via the base excision repair (BER) pathway^8^. Under thymineless conditions, reintroduction of uracil has been thought to result in futile repair cycles, eventually leading to cell death. Such a model suggests that manipulation of uracil excision would affect the efficacy of TYMS-inhibiting agents. However, despite a considerable amount of research, this remains elusive^12–22^.

In addition to post-replicative uracil removal, UNG2 excises mutagenic uracil from deaminated cytosine in single-stranded DNA (ssDNA) at replication forks, orchestrated by RPA^11,23^. However, further processing by AP endonucleases at the replication fork may lead to double-strand breaks (DSBs). To prevent this, HMCES binds to AP sites in ssDNA, forming a protein-DNA adduct that prevents strand cleavage, arrests replication and delays repair until the double-stranded conformation is restored by fork reversal^23,24^. HMCES knockout increased resistance to PMX^25^, likely due to a shift from replication arrest and fork reversal to replicative bypass of AP sites by DNA polymerase eta (POLH).

Histone acetylation is a crucial part of the epigenetic landscape and is regulated by histone acetyltransferases (HATs) and histone deacetylases (HDACs). HDACs may target histones, resulting in a transcriptionally repressed chromatin state and contribute to carcinogenesis by inhibiting transcription of tumor suppressor genes^26^. HDACs also target non-histone proteins implicated in cancer and various other diseases. Currently, five HDAC inhibitors (HDACis) are approved for the treatment of hematological malignancies. In addition, there are several ongoing clinical studies of HDACis in solid cancers, including NSCLC, primarily in combinatorial treatments and to resensitize tumors to standard therapy (www.clinicaltrials.gov).

Our group recently showed that HDACis reduce UNG2 levels via proteasomal degradation^27^. In this study, we further aimed to investigate the cytotoxic mechanisms of PMX and HDACi mono- and combination treatments, including the role of genomic uracil induction/processing and other DNA damage/repair mechanisms.

## Results

### HDACi decreases PMX sensitivity of NSCLC cells when added prior to PMX

**Fig. 1:**
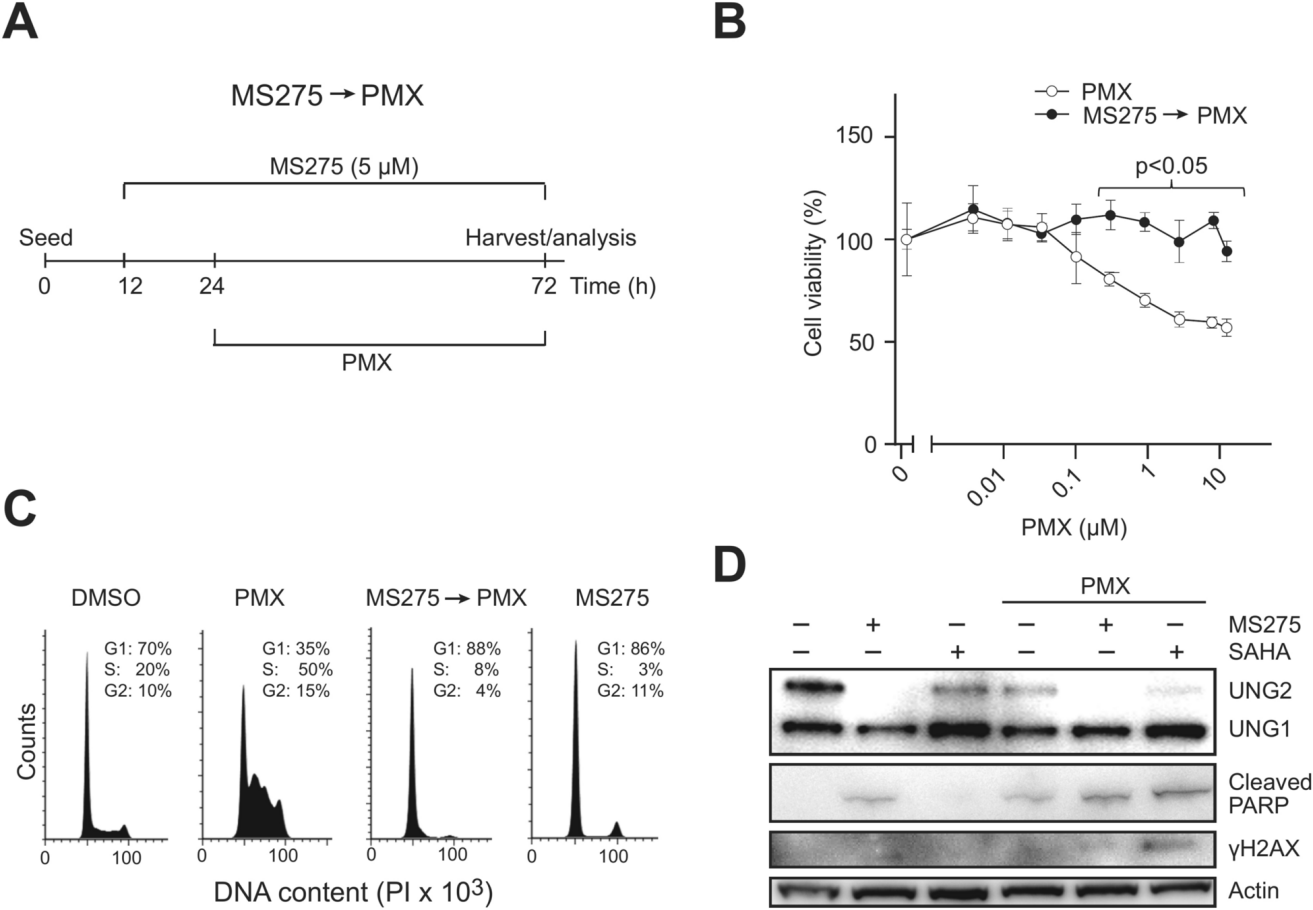
Treatment with HDACis prior to PMX mediates reduced PMX sensitivity. (**A**) Treatment schedule; A549 cells were pre-treated with 5 µM MS275 for 12 h prior to treatment with various concentrations of PMX for 48 h, as indicated in Fig. 1B (0.5 µM PMX was used in C and D). (**B**) Resazurin cell viability assays demonstrated that MS275 pretreatment mediated reduced sensitivity towards PMX. Each data point represents an average of three parallel wells relative to untreated wells with standard deviation indicated. (**C**) Flow cytometry analysis of cell cycle distribution after the various treatments at. (**D**) Western analysis of A549 whole cell extracts after the various treatments, including treatment with SAHA (60 h) that mediates less robust UNG2 depletion than MS275. Full western blots related to Fig. 1D are provided in Supplementary Figure S2.

**Fig. 2:**
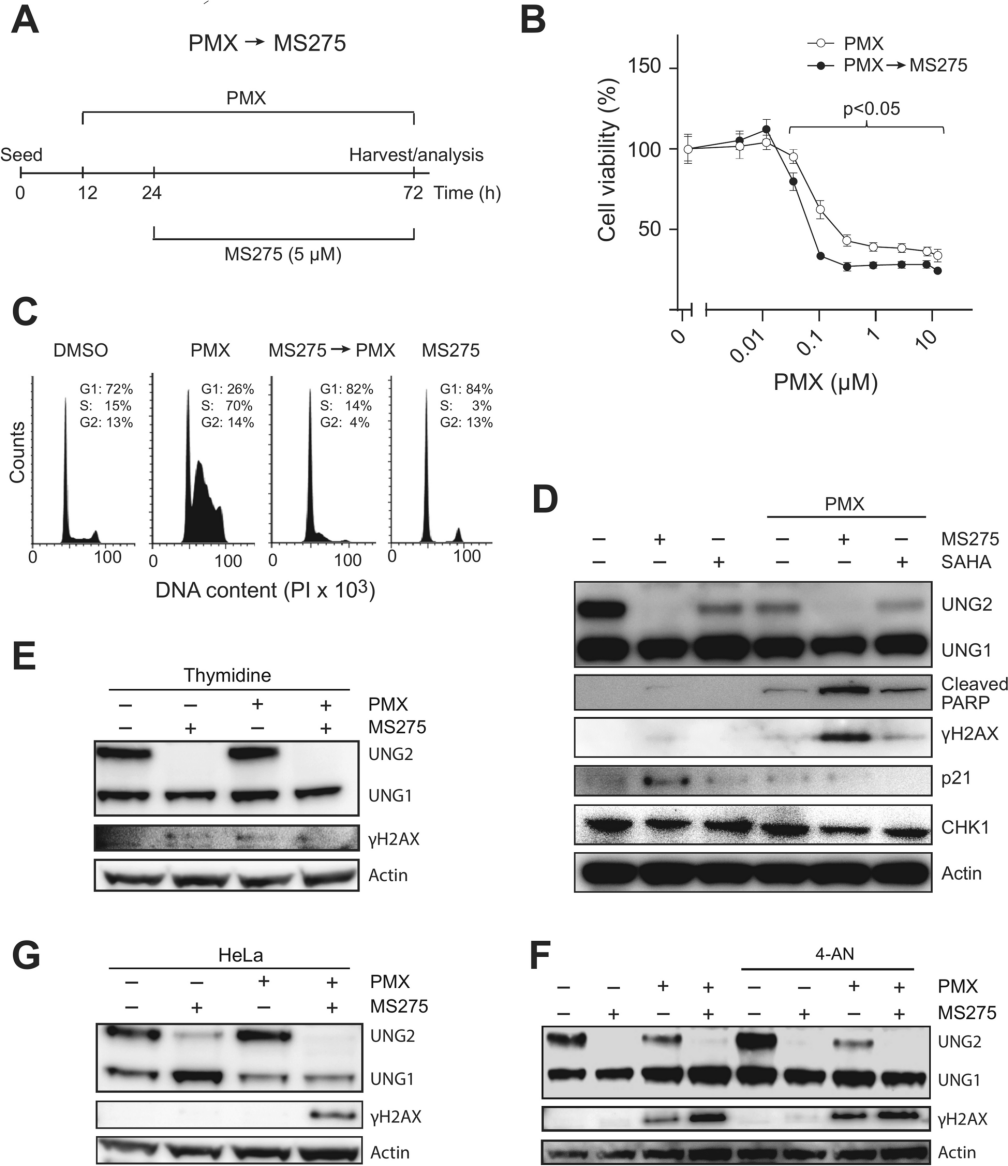
Treatment with HDACis after PMX mediates moderately increased PMX sensitivity. (**A**) Treatment schedule; A549 cells were pre-treated with various concentrations of PMX, as indicated in Fig. 2B (0.5 µM PMX was used in (**C**–**G**) for 12 h prior to addition 5 µM MS275 and further incubation for 48 h. (**B**) The reversed treatment schedule mediated moderately increased sensitivity towards MS275 as demonstrated by resazurin viability assays. Each data point represents an average of three parallel wells relative to untreated wells with standard deviation indicated. (**C**) Flow cytometry analysis of cell cycle distribution after the various treatments. (**D**) Western analysis of A549 whole cell extracts after the various treatments, including treatment with SAHA (48 h) that mediates less robust UNG2 depletion than MS275. (**E**) Addition of the PMX antidote thymidine (5 µM) attenuated the activation of γH2AX after PMX and PMX → MS275 treatment and increased expression of UNG2 in PMX-treated cells to the same level as in untreated cells. (**F**) Addition of the PARP inhibitor 4-AN (10 µM) together with PMX did not significantly affect the levels of UNG2 or γH2AX (**G**) PMX → MS275 treatment mediated robust induction of γH2AX in p53-deficient HeLa cells. Full western blots related to Fig. 2D are provided in Supplementary Figure S2, and full western blots related to Fig. 2E, F and G are provided in Supplementary Figure S3.

To optimize PMX sensitivity assays, A549 NSCLC cells were first cultured in ATCC-recommended medium (Ham’s F12K with 10% FBS). However, no significant viability reduction was observed even at PMX concentrations over 10 µM, likely due to the presence of ~ 3 µM thymidine in the medium and variable thymidine levels in FBS. To avoid these sources of dTTP, cells were cultured in DMEM (thymidine free) supplemented with dialyzed FBS. Under these conditions, we reproducibly observed IC_50_ values for PMX between 0.2 and 1 µM, depending on the cell density at the onset of PMX treatment and the duration of treatment (Fig. 1A, B, Fig. 2A, B).

HDACis induce proteasome-mediated degradation of UNG2 after 9–12 h of treatment in various cell lines^27^. To test whether HDACi-induced depletion of UNG2 affected sensitivity to PMX, A549 cells were treated with 5 µM MS275 (IC_25_) for 12 h followed by various concentrations of PMX (treatment schedule outlined in Fig. 1A). MS275 pretreatment significantly reduced PMX sensitivity at concentrations above 0.05 µM (Fig. 1B). PMX alone caused pronounced S-phase arrest (Fig. 1C), consistent with previous studies^3^. This was absent in MS275-pretreated cells (MS275 → PMX), apparently since MS275 induces a potent G1/S block. This suggests that PMX-induced cytotoxicity is linked to DNA replication stress, which is alleviated by MS275 blocking S-phase entry.

Western analysis demonstrated robust UNG2 depletion after MS275 and combined MS275 → PMX treatments (Fig. 1D). A less complete depletion was observed with SAHA, likely due to its much shorter half-life^28,29^. PMX alone also partially depleted UNG2 (Fig. 1D), likely because of accumulation of cells in S/G2 (Fig. 1C), where UNG2 levels are naturally low^30^. Notably, PMX-induced, cell cycle-independent UNG2 degradation has been reported in some cell lines^14^. PARP cleavage was weakly induced by MS275 and PMX individually and increased slightly with combined treatments, but no significant double-strand break (DSB) induction was observed, as indicated by γH2AX levels.

### HDACi increases PMX sensitivity of NSCLC cells when added after PMX

Reversing the treatment order, administering PMX first, followed by 5 µM MS275 (Fig. 2A), abolished the protective effect of HDACi and moderately increased PMX sensitivity at higher concentrations (Fig. 2B). The cell cycle distribution (Fig. 2C) and Western blot results reflected those from the previous schedule (MS275 → PMX), with MS275 depleting UNG2 and SAHA causing partial depletion (Fig. 2D). Notably, the PMX → MS275 treatment schedule induced significant PARP cleavage and robust γH2AX induction. These markers were also increased after combined PMX → SAHA treatment, although to a lesser extent.

Adding thymidine (dT, 5 µM), attenuated γH2AX activation after PMX and PMX → MS275 treatments (Fig. 2E), underscoring the role of low dTTP levels in cytotoxicity. Although thymidine prevented PMX-induced reduction of UNG2, it had no impact on MS275-mediated UNG2 depletion. PARP inhibition by 4-AN had no significant impact on γH2AX levels (Fig. 2F), suggesting that PMX- and PMX → MS275-induced DSBs are independent of UNG2-induced BER.

A previous study demonstrated that UNG knockdown increased PMX sensitivity in A549 cells sevenfold^18^, and that this was dependent on functional p53^22^. However, in p53-deficient HeLa cells, PMX → MS275 treatment still triggered UNG2 depletion and robust γH2AX activation, indicating the response is p53-independent.

### PMX sensitivity is unaffected by CRISPR/Cas9-mediated UNG depletion

**Fig. 3:**
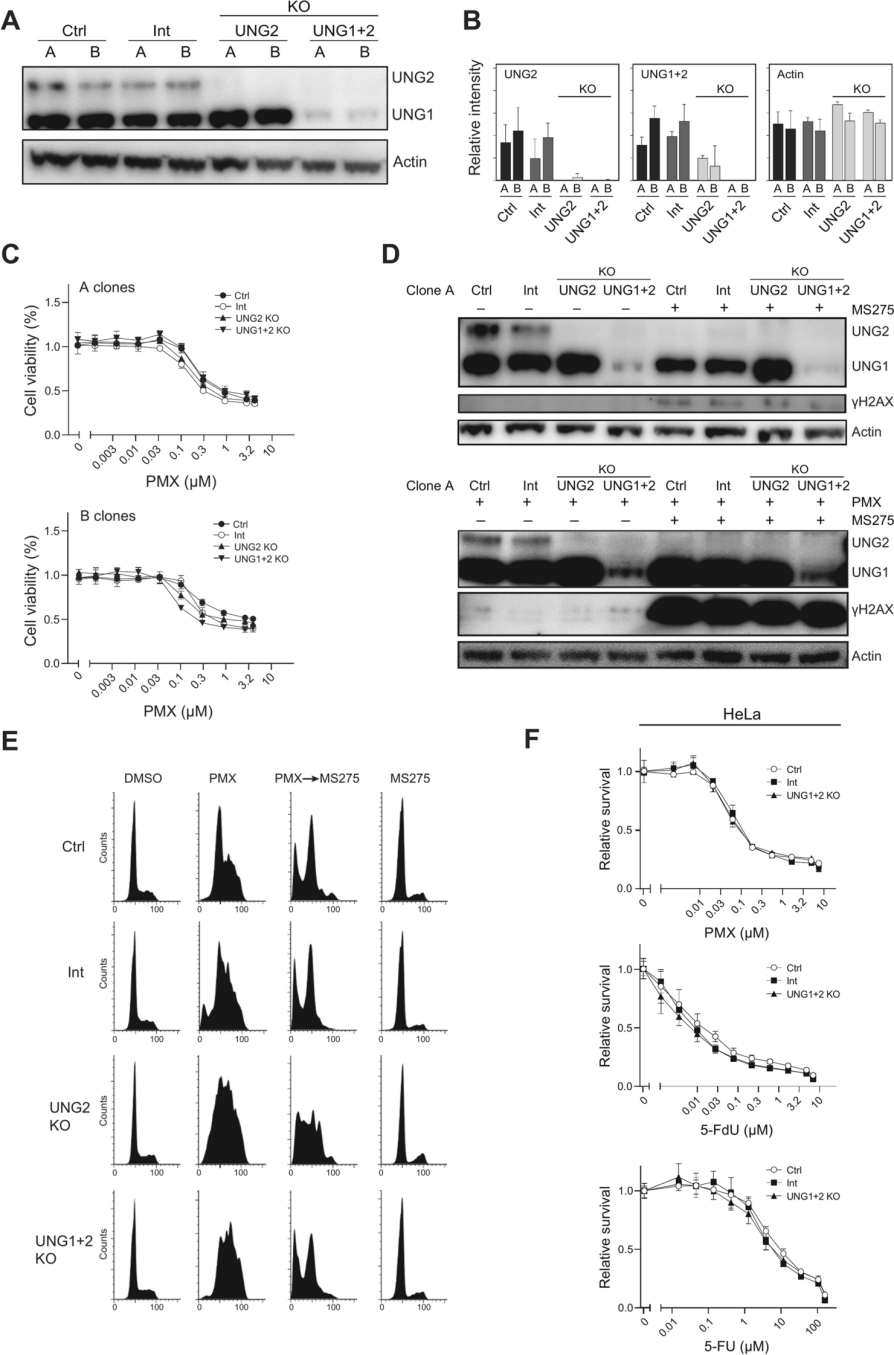
Depletion of UNG does not affect sensitivity towards PMX or fluoropyrimidines. A549 UNG2 KO, UNG1 + 2 KO and control cells transduced with virus without gRNA (Ctrl) or rRNA targeting Intron 2 in UNG (Int) were subjected to (**A**) Western analysis and (**B**) PRM targeted MS analysis of UNG and actin. (**C**) Parallel clones A and B of the A549 KO cells were subjected to PMX sensitivity analysis using resazurin assay. Each data point represents an average of three parallel wells relative to untreated wells with standard deviation indicated. (**D**) Upper panel: Clone A UNG KO A549 cells was subjected to 5 µM MS275 for 48 h prior to western analysis of UNG and γH2AX with actin as loading control. Lower panel: Clone A UNG KO A549 cells were pre-treated with 0.2 µM PMX for 12 h prior to addition of 5 µM MS275 for 48 h and subjected to western analysis of UNG and γH2AX with actin as loading control. (**E**) Cell cycle distributions were analyzed in parallel with flow cytometry. (**F**) Hela UNG1+2 KO cells and control cell lines were subjected to PMX, 5-FdU and 5-FU sensitivity analysis using resazurin assay. Full western blots related to Fig. 3A and 3D are provided in Supplementary Figure S2.

To further investigate the role of UNG in PMX and HDACi sensitivity, we used CRISPR/Cas9 to generate A549 UNG2 single- and UNG1 + 2 double knockout (KO) cells (Fig. 3A). Targeted PRM mass spectrometry^31^ verified the absence or strong reduction of UNG2 peptides in the single- and double-KO clones, while UNG1 peptides persisted in UNG2 KOs but were undetectable in double KOs (Fig. 3B). Additionally, we measured the expression of other proteins potentially affecting cytotoxicity of PMX (Supplementary Figure S1). No significant off-target effects were detected, validating the KO clones for further studies.

PMX sensitivity in the UNG KO clones was comparable to control clones (Ctrl and Int) (Fig. 3C). Western analysis showed no differences in γH2AX induction between the KOs and controls after PMX and/or MS275 treatment (Fig. 3D). Similarly, cell cycle distribution remained unchanged between the KOs and controls after these treatments (Fig. 3E), suggesting that neither UNG1 nor UNG2 significantly impacts PMX cytotoxicity or genotoxicity in A549 cells.

In p53-deficient HeLa cells, UNG1 + 2 KO did not affect sensitivity to PMX, 5-FU, or 5-FdU compared to controls (Fig. 3F). These findings indicate that UNG does not influence sensitivity to TYMS inhibition in either p53-positive or p53-negative models. Overall, neither uracil processing by UNG nor BER intermediates contribute significantly to PMX-induced double-strand breaks and cytotoxicity, and MS275 likely enhances PMX sensitivity through mechanisms independent of UNG2 depletion.

### Most proteins affected by PMX and MS275 combinatorial treatment are downregulated

**Fig. 4:**
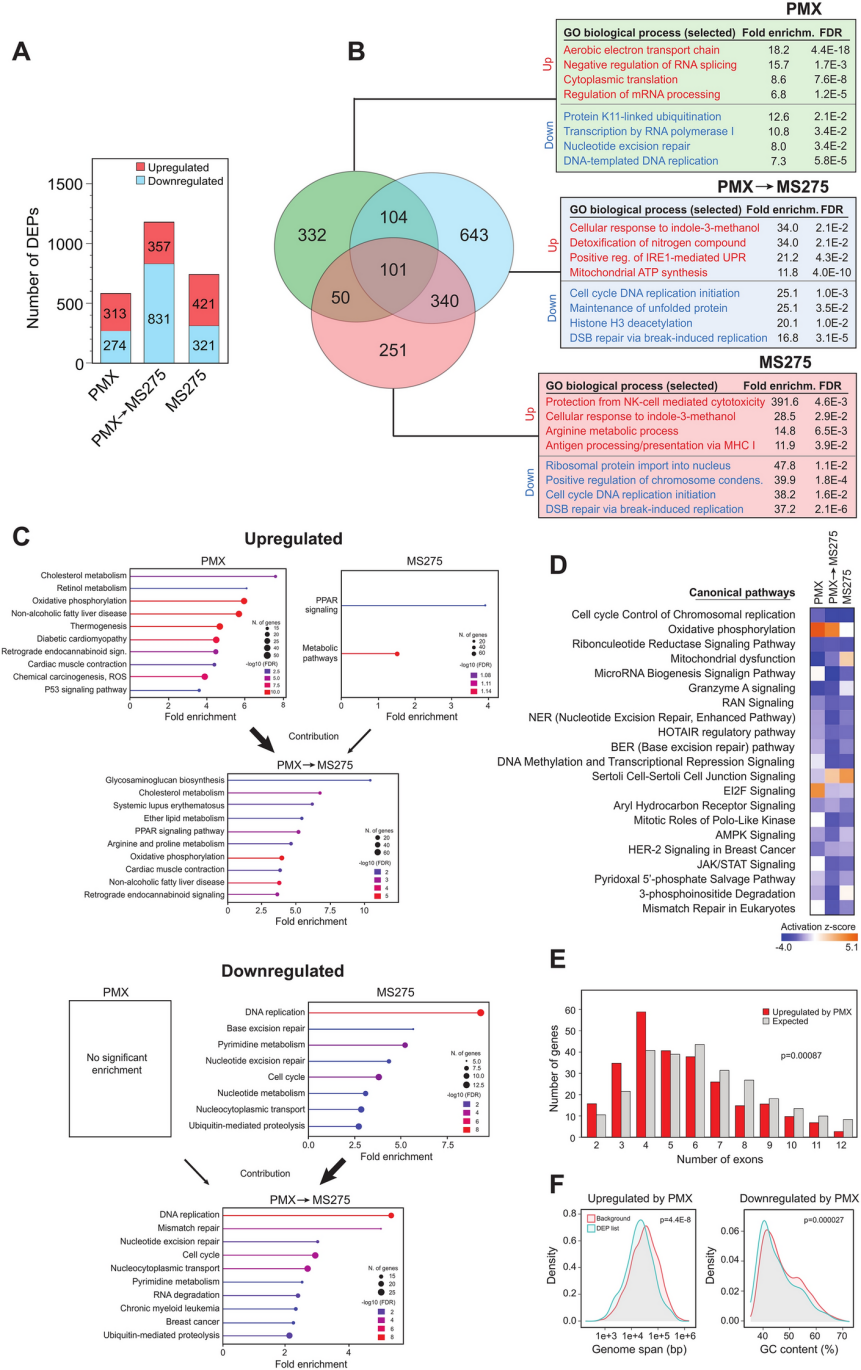
Differentially expressed proteins in A549 cells after single and combined PMX/MS275 treatments according to the protocol depicted in Fig. 2A, and whole cell proteomes subjected to LFQ MS/MS analysis (Supplementary Table S2). (**A**) The number of upregulated and downregulated proteins after each of the three treatment modalities. (**B**) The DEPs and their overlap after the different treatments are illustrated as a Venn diagram. The top affected GO biological processes from PANTHER analysis are indicated in the associated boxes. (**C**) Upregulated (upper panels) and downregulated (lower panels) KEGG pathways reported by ShinyGo analysis. Thick arrows indicate which of the single treatments that also mediate a dominant effect in the combined treatment. (**D**) Most affected canonical pathways reported from IPA analysis across the treatments. (**E**) ShinyGo analysis of proteins upregulated by PMX revealed a significant (Chi Square test) skewing towards proteins encoded by genes harboring few exons. (**F**) As in G, demonstrating significant skewing towards short genes among those upregulated by PMX (left) and towards higher AT-content among those downregulated by PMX (right).

To further investigate mechanisms underlying cytotoxicity of PMX and MS275 treatments, we performed LFQ MS on A549 cells, untreated or treated with PMX, MS275, or PMX → MS275 (Fig. 2A). We quantified 6666 protein groups across the samples (Control-6478; PMX-6538; MS275-6584; PMX → MS275-6414, Supplementary Table S2). Differentially expressed proteins (DEPs, p < 0.05, absolute log2 LFQ difference >|0.585|, corresponding to 1.5-fold change) were selected for further analysis. DEPs also included proteins quantified exclusively in either control or treatment groups, represented by log_2_ difference ratios of −100 or 100. The filtering identified 587 (PMX), 1188 (PMX → MS275) and 742 (MS275) DEPs after the different treatments. Notably, while the majority of DEPs were upregulated in single treatments with PMX (53%) or MS275 (57%), most DEPs (70%) were downregulated in the combined PMX → MS275 treatment (Fig. 4A). The mechanisms driving this shift in protein regulation remain to be elucidated.

### PMX upregulates mitochondrial electron transport and downregulates DNA replication and RNA splicing

Analysis of differentially expressed proteins (DEPs) using various bioinformatics tools revealed that PMX treatment significantly upregulates the aerobic electron transport chain (ETC) (Fig. 4B-D). In total, 22 proteins from ETC complexes I to IV were upregulated by PMX, including 12 subunits of the NAD dehydrogenase complex. Most of these were also upregulated in PMX → MS275. Interestingly, of the 12 quantified subunits of the mitochondrial ATPase (complex V), only two were upregulated by PMX, and none were upregulated in PMX → MS275. This suggests an imbalance in oxidative phosphorylation, leading to increased mitochondrial membrane potential. Supporting this, the mitochondrial ATPase inhibitor ATPIF1 was markedly upregulated in both PMX (5.5-fold) and PMX → MS275 (3.2-fold). Increased ETC activity relative to ATP synthase likely enhances ROS production, consistent with previously reported elevated ROS levels in PMX-treated lung cancer cells^32^.

All treatments negatively impacted DNA replication, with core subunits of DNA polymerases α, β, and δ significantly downregulated. (> 2.5-fold). Initiating polymerase subunits POLA1 and POLA2 were most affected (8.6- and 15-fold downregulated in PMX → MS275). Among proteins controlling the cell cycle, the levels of CDK1, CDK6, CDK12, and CyclinB1 (CCNB1) were unaffected by PMX, but markedly downregulated in MS275 and PMX → MS275. Conversely, p21 (CDKN1A), a canonical HDAC1/2 target, was markedly upregulated (6.3–9.3-fold) in all three treatments.

The second most affected GO process by PMX was negative regulation of RNA splicing. Altered splicing may be reflected in the average number of exons in the genes encoding upregulated proteins after PMX treatment, which was very significantly skewed towards fewer exons (Chi-squared test p = 0.00087) (Fig. 4E). The genes encoding proteins that were upregulated by PMX also spanned significantly shorter genome lengths than the overall background (Fig. 4F, left). Potentially contributing to this could be that longer mRNAs are more efficiently localized to stress granules during stress and are not translated^33^. Finally, we observed that proteins downregulated by PMX have a significantly lower GC (and consequently higher AT) content compared to the average in our dataset (Fig. 4F, right). This may be partly due to the selective misincorporation of dUMP in AT-rich genes. Misincorporated uracil has been shown to negatively impact gene expression, not by directly blocking transcription, but through repair intermediates generated by UNG-mediated uracil excision^34^. Consistent with this, we did not observe a bias towards AT content following PMX → MS275 treatment, where UNG2 is depleted.

### Proteins involved in purine and pyrimidine metabolism are differentially affected by PMX and MS275

Proteins involved in purine and pyrimidine metabolism showed differential responses to PMX and MS275 treatments. The primary target of PMX, TYMS, was 4.4-fold upregulated by PMX, potentially as a compensatory response to dTTP depletion. In contrast, TYMS was threefold downregulated by PMX → MS275 and 32-fold by MS275 (Supplementary Table S2). MS275 thus contributes to overall reduction of TYMS activity in the combinatorial treatment. Notably, the ribonucleotide reductase subunits RRM1 and RRM2 remained unaffected by PMX alone but were strongly downregulated (6.1- and 28-fold) by PMX → MS275, which may be clinically significant given their correlation with poor pemetrexed/gemcitabine response^35^.

Nucleotide pool sanitation proteins ASMTL and SAMHD1 were upregulated by MS275. ASMTL, which was also markedly upregulated (9.4-fold) in PMX → MS275, has nucleoside triphosphate pyrophosphatase activity against several methylated ribonucleotides, 8-oxoGTP, UTP and dTTP^36^ and may thus, in combination with PMX, contribute to dTTP depletion. SAMHD1, the only known dNTP triphosphohydrolase in eukaryotes and a poor prognostic factor in NSCLC^37^, regulates dNTP pools and aids in degradation of nascent DNA at stalled replication forks^38^.

### Pemetrexed and MS275 mediate downregulation of DNA repair and genome maintenance proteins

**Fig. 5:**
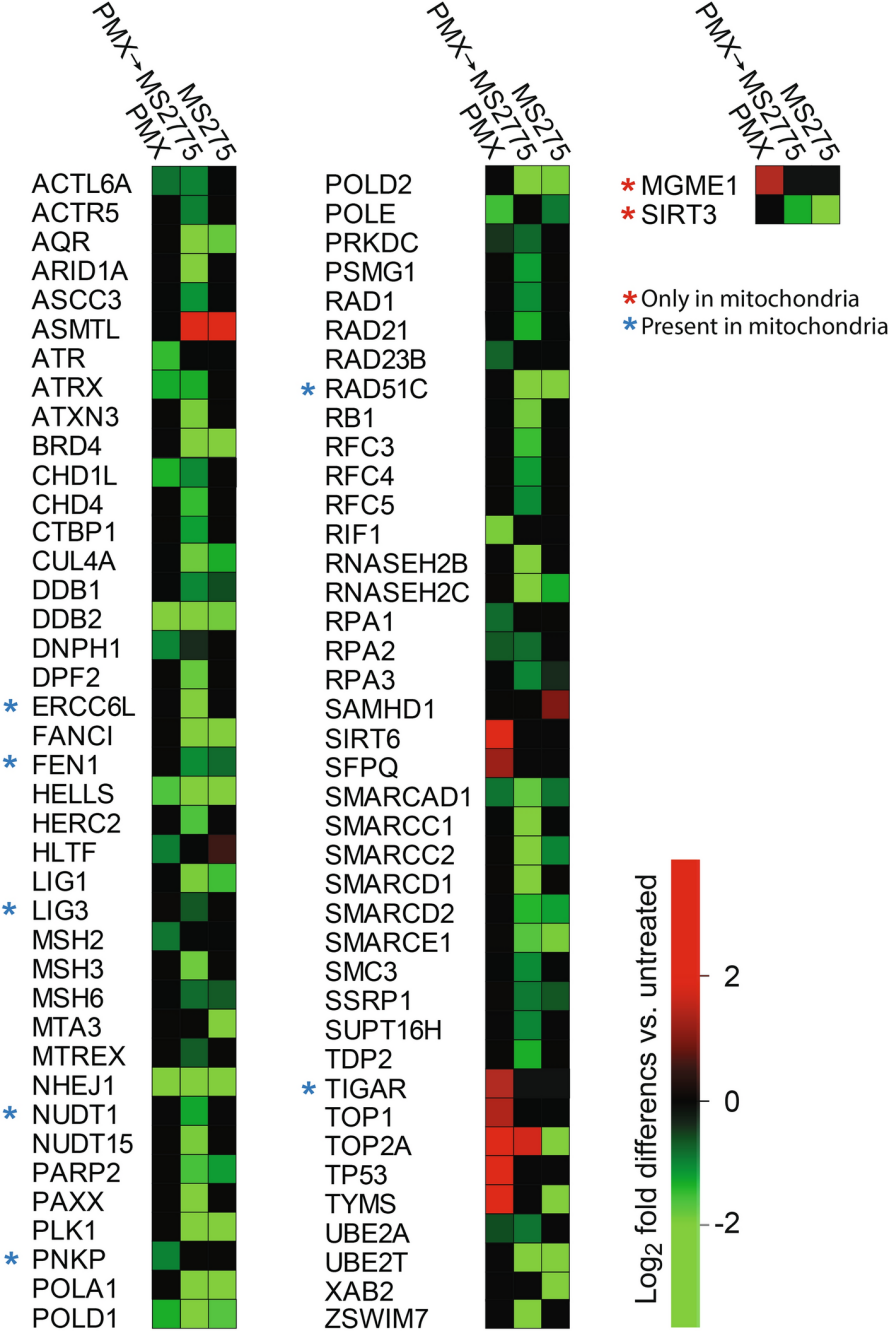
PMX → MS275 mediated overall downregulation of known DNA repair proteins (Supplementary Table S2). Graphic representation of DNA repair proteins significantly affected (> + / − 1.5-fold, p < 0.05) by at least one of the treatments (MS275, PMX or PMX → MS275). Proteins present in both nuclei and mitochondria are denoted with a blue asterisk, whereas those only found in mitochondria are denoted with a red asterisk.

Enhanced DNA repair has previously been suggested to mediate PMX resistance in NSCLC^5^. A manual comparison of differentially expressed proteins (DEPs) revealed that 19 proteins were downregulated by PMX, 30 by MS275, and 60 by the combined PMX → MS275 treatment (Fig. 5). Many of these proteins are involved in crucial DNA repair pathways, such as base excision repair (BER), mismatch repair (MMR), and homologous recombination (HR). Notably, UNG was reported not to be significantly affected in the LFQ analyses, since UNG1 remains largely unaffected by the treatments, and contributes to the total UNG quantification by sharing all its detected tryptic peptides with UNG2.

Many DNA repair proteins are cell cycle regulated, but treatment-induced cell cycle change was apparently not a major contributed to their observed altered expression. For example, many proteins that display peak expression in S phase^39^, like FEN1, LIG1 and MSH6, were significantly downregulated even after PMX treatment, which strongly enriched cells in S phase. Conversely, TOP2A, which is highly expressed in G2, was upregulated following PMX → MS275 treatment, despite a marked reduction in the number of cells in G2 phase (Fig. 2C).

In addition to BER, mismatch repair (MMR) contributes to sanitation of genomic uracil, and enhanced levels of MSH2 has previously been implicated in PMX resistance^40^. Both MSH3 and MSH6 were significantly downregulated in PMX → MS275, suggesting reduced overall MMR. Moreover, RNaseH2 subunits B and C, involved in ribonucleotide excision repair (RER), were profoundly downregulated by PMX → MS275, (4.8- and 3.9-fold, respectively), suggesting impaired removal of misincorporated ribonucleotides. Our data also suggest that MS275 downregulates homologous recombination (HR) repair. Both PARP2, which stimulates end resection at DSBs, and the essential HR factor RAD51C, were markedly downregulated after MS275 and PMX → MS275 treatments. In addition, PLK1, a regulator of RAD51 and HR, was strongly downregulated by MS275-containing treatments (22–26-fold).

Finally, RAD51 is recruited to stalled replication forks by the FANCI-FANCD2 (D2-I) complex, which also protects forks from unscheduled degradation^41^. FANCI was significantly downregulated following MS275 (5.6-fold) and even more so with PMX → MS275 (9.7-fold), potentially increasing the risk of stalled replication forks being converted into double-strand breaks (DSBs).

A subset of DNA repair proteins was significantly upregulated by PMX alone but remained largely unaffected after combinatorial treatment. Of these, TIGAR, which stimulates the pentose phosphate pathway and thereby enhances NADPH and resistance to oxidative stress, has been correlated with high metastatic potential of NSCLC cell lines^42^. TOP1 is part of an alternative and mutagenic repair pathway for misincorporated ribonucleotides in DNA in the absence of RNaseH2-induced RER^43^. Such a pathway could be especially relevant after PMX → MS275 treatment since this mediated significant downregulation of RNaseH2 subunits RNASEH2B/C (Fig. 5). Finally, TP53 was robustly induced by PMX alone, but additional MS275 treatment rendered TP53 below the detection limit.

Several chromatin remodeling proteins that regulate DNA repair, particularly subunits of the BAF (SWI/SNF) complex, were also markedly downregulated in MS275-containing treatments. Loss of BAF function has been linked to mismatch repair deficiency^44^ and increased tumor mutational burden in NSCLC^45^, which might contribute to altered tumor responses to therapies.

These findings collectively suggest that PMX and MS275 suppress critical DNA repair mechanisms, leading to enhanced genomic instability and potentially increasing the efficacy of chemotherapy in NSCLC.

### Targeted mass spectrometry corroborates the results from LFQ analysis

**Fig. 6:**
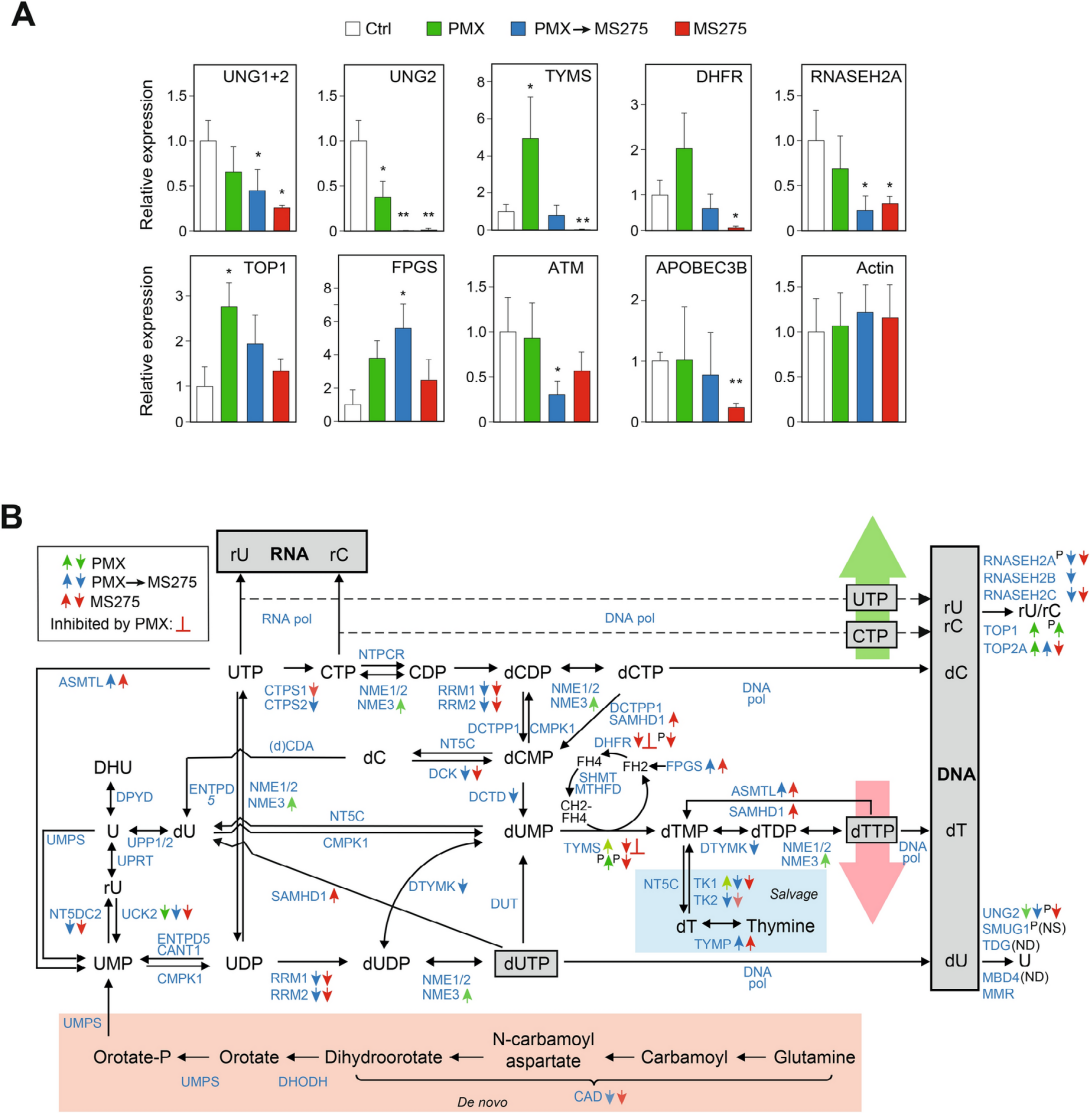
PMX and MS275 mediates differential expression of several proteins in pyrimidine synthesis and DNA repair, highlighting misincorporation of ribonucleotides in DNA as a potential mediator of cytotoxicity of PMX. (**A**) Total cellular proteins from untreated A549 cells and cells subjected to PMX, MS275 or PMX → MS275 treatment according to the schedule depicted in Fig. 2A and subjected to targeted PRM analysis of indicated proteins. Expression levels are given relative to untreated cells. *t*-test p-values < 0.05 is denoted by * and p < 0.01 by **. (**B**) Overview of pyrimidine metabolism and DNA repair proteins involved in the initial processing of nucleotides misincorporated during DNA synthesis. Targets of PMX are indicated (red _┴_) along with differently colored arrows which show treatment specific DEPs identified in LFQ and PRM (the latter indicated with preceding P) analyses. ASMTL probable bifunctional dTTP/UTP pyrophosphatase/methyltransferase protein, CAD multifunctional protein CAD (carbamoylphosphate synthetase 2, aspartate transcarbamoylase, and dihydroorotase), CANT soluble calcium-activated nucleotidase, CDA cytidine deaminase, *CMPK* UMP-CMP kinase, CTPS CTP synthase, (d)CDA (deoxy)cytidine deaminase, DCK deoxycytidine kinase, DCTD dCMP deaminase, DCTPP dCTP pyrophosphatase, DHFR dihydrofolate reductase, DHODH dihydroorotate dehydrogenase, DPYD dihydropyrimidine dehydrogenase, DNA pol DNA polymerase, dT thymidine, DTYMK thymidylate kinase, DUT deoxyuridine 5ʹ-triphosphate nucleotidohydrolase, ENTPD nucleoside diphosphate phosphatase, FPGS folylpolyglutamate synthase, *MTHFD* C-1-tetrahydrofolate synthase, *NME* nucleoside diphosphate kinase, *NT5C* 5ʹ(3ʹ)-deoxyribonucleotidase, *NT5DC* 5ʹ-nucleotidase domain-containing protein, *NTPCR* cancer-related nucleoside-triphosphatase, RRM ribonucleotide reductase, SAMHD deoxynucleoside triphosphate triphosphohydrolase, SHMT serine hydroxymethyltransferase, TK thymidylate kinase, TYMP thymidine phosphorylase, TYMS thymidylate synthase, UCK uridine-cytidine kinase, UMPS uridine 5ʹ-monophosphate synthase, UPP uridine phosphorylase, UPRT uracil phosphoribosyltransferase homolog.

To validate the LFQ proteomics findings, we conducted targeted PRM-MS/MS analysis of select proteins (Supplementary Table S1, Fig. 6A). The PRM data corroborated > 98% reduction in UNG2 following MS275 and PMX → MS275 treatments, matching the degradation pattern observed in western analysis. Additionally, MS275-induced downregulation of TYMS (30-fold) and DHFR (11-fold) in PRM aligned with LFQ results (32- and 13-fold, respectively). RNASEH2A, although not significantly downregulated in the LFQ analysis for MS275 regimens, was significantly reduced in both treatments by PRM, confirming RNaseH2 heterotrimer downregulation. TOP1 was reported as 2.7-fold upregulated by PMX, in agreement with the 2.5-fold upregulation reported in LFQ. FPGS, which catalyzes polyglutamation of folates and antifolate drugs, thereby mediating their intracellular retention and affinity for other folate enzymes, was markedly upregulated in PMX → MS275 treatment (Fig. 6A). Loss of FPGS activity has previously been associated with resistance to the TYMS inhibitor methotrexate in several leukemia cell lines^46^ and FPGS protein expression is associated with PMX response in malignant pleural mesothelioma^47^. Furthermore, a single nucleotide polymorphism that enhanced FPGS expression in NSCLC cell lines is associated with a higher response rate in patients treated with PMX and platinum drugs^48^.

ATM was not quantified by LFQ, but 3.3-fold downregulated by PMX → MS275 in PRM. Given ATM’s importance in DNA damage response^49^ and its mutations in 10% of NSCLC tumors^50^, this could have clinical implications for immunotherapy responses^50^. Finally, APOBEC3B, also absent in LFQ but downregulated 4.2-fold by MS275 in PRM, has been linked to resistance against EGFR-targeted therapies in NSCLC^51^. Further evaluation of HDACi-mediated APOBEC3B downregulation to avoid acquired resistance to EGFR inhibitors is thus warranted.

In summary, the collective MS analyses indicate that MS275 alters the expression of proteins involved in pyrimidine metabolism, thereby enhancing PMX-induced depletion of dTTP and misincorporation of uracil-containing nucleobases during replication. A concomitant depletion of DNA repair proteins involved processing of these lesions likely contributes to increased sensitivity towards PMX.

## Discussion

In this study, we observed schedule-dependent modulation of PMX cytotoxicity when combined with the HDACi MS275, which aligns with prior findings showing that HDAC inhibitors like Givinostat can reverse PMX resistance in NSCLC cell lines, regardless of p53 status^52,53^. Our CRISPR/Cas9 knockout experiments indicated that PMX cytotoxicity is largely independent of UNG2 and its role in processing dUMP incorporation, consistent with minimal effects observed in similar studies with RTX and UNG inhibition^13^.

Proteomic analysis revealed numerous differentially expressed proteins (DEPs) associated with pyrimidine metabolism and DNA repair, many of which have not previously been linked to PMX or MS275 treatment. Figure 6B illustrates the DEPs involved in pyrimidine metabolism and processing of misincorporated nucleotides in DNA. From this, it is evident that the MS275-mediated downregulation of RRM1, RRM2 and CAD should decrease dUTP levels and consequently reduce dTTP generated via de novo synthesis. PMX → MS275 also mediated a twofold reduction of DCDT, which deaminates dCMP to the TYMS substrate dUMP. Additionally, MS275 negatively affected the salvage pathway of dTTP synthesis by downregulating the rate-limiting thymidine kinase (TK). The strong upregulation of TYMP (33- and 25-fold by MS275 and PMX → MS275, respectively) could be relevant in capecitabine treatment, which is metabolized into its active form, 5-FU, by TYMP. Consistent with this, SAHA has been demonstrated to synergize with capecitabine by upregulating TYMP^54^. Finally, ASMTL, which dephosphorylates dTTP to dTMP, was markedly upregulated in MS275 and PMX → MS275.

Overall, the MS275-induced alterations in pyrimidine metabolism are expected to enhance PMX’s cytotoxic effects by further depleting dTTP, making this combination a promising therapeutic strategy.

During TYMS inhibition, the loss of dTTP increases the dUTP/dTTP and UTP/dTTP ratios, leading to misincorporation of both dUMP and the ribonucleotide UMP into DNA. Replicative polymerases, particularly POLE, have incomplete discrimination against ribonucleotides, incorporating substantial NMPs, which may outnumber all other DNA lesions^55^. POLE also has the lowest discrimination against UTP^56^, leading to markedly increased misincorporation of UMP after PMX treatment. After PMX → MS275 treatment, further increases in NMP incorporation may occur due to the downregulation of key enzymes involved in nucleotide synthesis, such as RRM1/2 and TK1/2. Ribonucleotides incorporated into DNA are repaired via RNaseH2-mediated ribonucleotide excision repair (RER), but the significant depletion of RNASEH2 and other RER components such as FEN1 and LIG1 suggests this pathway is downregulated by MS275, leading to NMP accumulation. The latter is supported by experiments in RNAseH2-deficient yeast^57,58^.

Incorporated ribonucleotides may act as sensors for nucleotide pool imbalances, triggering replication stress and ATR activation^59,60^. This conforms to the increased survival of cells treated with MS275 prior to PMX, since MS275 blocks S-phase entry. Unrepaired ribonucleotides can cause SSBs and DSBs^61^, consistent with the elevated γH2AX levels seen in PMX → MS275-treated cells. In the absence of RER, ribonucleotides can be subject to error-prone TOP1 processing, leading to genomic instability^43^. Finally, several lines of evidence indicate that genomic ribonucleotides can activate the cGAS-STING pathway^62,63^, potentially enhancing immune responses to checkpoint inhibitors.

While the exact role of TOP1 in PMX → MS275 cytotoxicity remains unclear, TOP1 binds to UMP but not to dUMP in DNA, and PMX and RTX induce TOP1 trapping lesions^64^. These lesions are typically resolved by TDP1 or NER pathways involving ERCC1, XPF and RPA^65^. Low ERCC1 expression has been associated with prolonged survival in lung adenocarcinoma patients treated with PMX as first line therapy^66^. A novel DDX3X-mediated NMP removal pathway has been reported, though its contribution to cytotoxicity in vivo requires further investigation^67^.

The effects of misincorporated ribonucleotides in mtDNA deserve special consideration since mitochondria lack RNaseH2. The extent to which mitochondrial TOP1MT resolves incorporated NMPs is unknown, but the absence of typical 2–5 nucleotide deletions despite substantial levels of NMPs in mtDNA, suggests limited TOP1MT activity in processing these lesions^68^. The alkaline mitochondrial environment, driven by elevated ETC activity (observed after PMX treatment), may increase the susceptibility of NMPs in mtDNA to hydrolysis^69^. Furthermore, oxidative stress induced by enhanced ETC activity could exacerbate mtDNA damage. Notably, PMX downregulates PNKP, critical for repairing oxidized bases in both nuclear and mitochondrial contexts, while MS275 depletes RAD51C, impeding mitochondrial HR^70^. This highlights a potential role for RAD51C in PMX-induced cytotoxicity, supported by findings that Givinostat resensitizes PMX-resistant cells through RAD51 downregulation^52^.

**Fig. 7:**
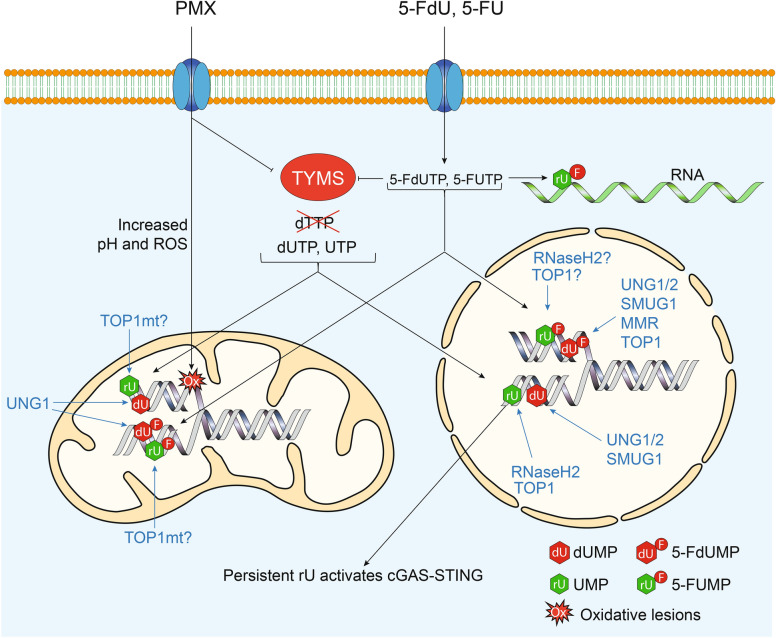
Model for cytotoxic mechanism of PMX and fluoropyrimidines (5-FdU and 5-FU). TYMS inhibition results in reduced dTTP and increased relative levels of dUTP and rUTP which are incorporated during DNA replication in both mitochondrial and nuclear DNA, forming dU (red) and rU (green) lesions, respectively. PMX mediates oxidative damage while 5-FUMP is incorporated into RNA. The fluorinated deoxy- and the ribonucleotide analogues are also incorporated into mitochondrial and nuclear DNA, as 5-FdUMP and 5-FUMP, respectively. DNA repair proteins or pathways (in blue) excise their indicated DNA lesions. Persistent rU in nuclear DNA may result in cGAS-STING activation.

Figure 7 illustrates our model for cytotoxicity induced by PMX and fluoropyrimidines. Nucleotide pool imbalance following TYMS inhibition leads to dUMP and UMP misincorporation into nuclear and mitochondrial DNA. Our data suggest that dUMP misincorporation and UNG-mediated processing play minor roles in cytotoxicity. However, ribonucleotide incorporation, especially UMP, may overwhelm the RER/TOP1-mediated repair mechanisms, resulting in DSBs. MS275 exacerbates this by depleting RNaseH2 and related repair proteins and by disrupting nucleotide pool regulation.

Fluoropyrimidine analogs are incorporated in the genome and mediate a nucleotide pool imbalance similar to that of antifolates. While genomic 5-FdUMP lesions may be repaired by UNG, MMR or TOP1^13,17,71^, our previous research suggests that UNG activity is not a significant contributor to 5-FU cytotoxicity^17^. Instead, 5-FU primarily exerts its effects through RNA misincorporation, though it can also be incorporated into DNA as 5-FUMP^72^.

To the best of our knowledge, it has not been investigated to what degree RNaseH2, TOP1 or other repair systems are able to remove 5-FUMP from DNA. Due to the interplay between MMR and RER, this may explain the dependency of 5-FU cytotoxicity on MMR proteins^73^.

In summary, our study identifies key pathways through which the HDAC1/3 inhibitor MS275 and PMX interact to enhance cytotoxicity in a schedule-dependent manner. MS275 exacerbates PMX-induced dTTP depletion by downregulating TYMS, DHFR, and FPGS. The depletion of UNG does not significantly contribute to the increased cytotoxicity observed with combined PMX → MS275 treatment. Instead, we propose that ribonucleotide misincorporation, especially UMP, plays a central role in PMX-induced DNA damage and cytotoxicity. MS275 likely aggravates this by inhibiting ribonucleotide repair in both nuclear and mitochondrial DNA.

## Methods

### Cell culture and preparation of extracts

HeLa and A549 (lung carcinoma) cell lines were purchased from the American Type Culture Collection (ATCC). HeLa cells were grown in Dulbecco’s modified Eagles medium, A549 was grown in Ham’s F12K and Dulbecco’s Modified Eagle Medium (DMEM), all media were supplemented with 10% fetal bovine serum (FBS), 2 mM L-glutamine, 2.5 µg/ml amphotericin B, 0.1 mg/ml gentamicin. Experiments were conducted with dialyzed FBS, if not indicated otherwise. FBS dialysis was performed overnight against PBS using a 6–8 kDa cutoff membrane. Cell lines were cultured at 37 °C at 5% CO_2_.

Whole cell extracts were prepared after trypsin detachment, washing twice with ice cold PBS and centrifugation at 0.4 × 10^3^ g for 5 min. Cell pellets were resuspended in 2 × PCV (packed cell volume) of lysis buffer (10 mM HEPES pH 7.9, 100 mM KCl, phosphatase inhibitor cocktail 2 and 3 (Sigma), Complete protease inhibitor (Roche), 10 µM MG132, 2 µM SAHA, and subjected to horizontal rolling for 1 h at 4 °C. Cells were then centrifuged for 10 min at 1.6 × 10^4^ g, and supernatants collected. Protein concentrations were measured by the Bradford assay (Bio-Rad) using bovine serum albumin as standard, and the extracts snap frozen in liquid nitrogen and stored at − 80 °C.

### Flow cytometric analysis of cell cycle

To analyze the cell cycle, cells were harvested and washed as described above, then fixed in ice-cold methanol at a concentration of 10^4^–10^5^ cells/ml. Following fixation, cells were washed with PBS and incubated with 200 µl RNaseA solution (100 µg/ml in PBS) at 37 °C for 30 min. DNA was then stained by adding 200 µl of propidium iodide (PI, 50 μg/ml, Sigma) and incubating at 37 °C for 30 min. Flow cytometry was conducted using a BD FACS Canto flow cytometer (BD Biosciences) with PI fluorescence excited by a 488 nm blue laser and detected in the phycoerythrin (PE) channel (578 nm). Data were analyzed using FlowJo^R^ version 10 software.

### Electrophoresis and western analysis

Protein extracts were prepared by dissolving them in NuPAGE LDS sample buffer (Thermo Fisher Scientific) and heating at 70 °C for 10 min. Proteins were separated by electrophoresis using 10% NuPage Novex Bis–Tris gels with MOPS running buffer. After electrophoresis, proteins were transferred to PVDF membranes (Immobilon) by electroblotting. Immunostaining was carried out at room temperature, beginning with a 1 h blocking step in PBST (PBS, 0.1% Tween) containing 5% nonfat dry milk. Membranes were then incubated with the primary antibody diluted in blocking buffer for 1 h. Following three 10-min washes in PBST, membranes were incubated with secondary antibodies diluted in in PBST for 1 h. For γH2AX antibody staining, TBS(T) was used in place of PBS(T). Membranes probed with HRP-conjugated secondary antibodies were washed three times for 10 min in PBST, treated with SuperSignal West Femto substrate (Thermo Fisher Scientific) and visualized using the Kodak Image station 4000R.

### Antibodies and reagents

Primary antibodies: MAb 2c12 (0.5 µg/ml) (OriGene TA503563) recognizing the common catalytic domain of UNG1/2, mAb beta-Actin (Abcam Ab8226), pAb p21 (abcam Ab18209), pAb γH2AX (Abcam ab2893), pAb cleaved PARP (Abcam Ab4830). Secondary antibodies: Swine anti-rabbit, HRP (Dako Chemicals), rabbit anti-mouse, HRP (Dako Chemicals), rabbit anti-goat, HRP (Dako Chemicals). Vorinostat (SAHA) (CAYMAN chemicals Cat#10009929), Entinostat (MS275) (Selleck S1053), Alimta/Pemetrexed (Active Biochem A-2002), 4-AN (Sigma A0966).

### Targeted quantitative proteomics

Whole cell extracts (50 µg protein) were treated with 5 mM tris(2-carboxyethyl)phosphine (TCEP) for 30 min to reduce disulfide bonds, followed by alkylation with iodoacetamide (1 µmol/mg protein) for 30 min in the dark. Proteins were precipitated using methanol–chloroform as previously described^74^, with a subsequent round of reduction and alkylation prior to overnight digestion with trypsin (Promega) at 1:50 enzyme-to protein ratio (w/w) at 37 °C. The resulting tryptic peptides were dried, resuspended in 0.1% formic acid (FA), and equal quantities from each sample were subjected to analysis on a QExactive mass spectrometer (Thermo Fisher Scientific) coupled to an Easy-nLC 1000 UHPLC system (Thermo Fisher Scientific /Proxeon). Samples (2 µg peptides) were injected onto an Acclaim PepMap100 C-18 trap column (75 µm i.d. × 2 cm, C18, 3 µm particle size, 100 Å pore size) (Thermo Fisher Scientific) and separated on an Acclaim PepMap100 C-18 analytical column (75 µm i.d. × 50 cm, C18, 2 µm particle size, 100 Å pore size) (Thermo Fisher Scientific). Peptides were resolved using a 115 min gradient at a flow rate of 250 nl/min, starting with 100% buffer A (0.1% FA) increasing to 5% buffer B (100% Acetonitrile, 0.1% FA) within 2 min, then gradually to 35% buffer B over 98 min, followed by a rapid rise to 100% buffer B over 6 min, and then held for 9 min. Eluting peptides were ionized via a nanospray ESI ion source (Proxeon, Odense) and analyzed in positive ion mode using 1.9 kV electrospray voltage and HCD fragmentation. MS/MS scans were performed at 35 000 FWHM resolution, using a normalized collision energy (NCE) of 28, automatic gain control (AGC) target of 2 × 10^5^, maximum injection time of 120 ms, and a 2 m/z isolation window. Targeted parallel reaction monitoring (PRM) methods were designed, analyzed, and conducted in Skyline software (v3.1.0.7382)^75^. Proteotypic peptides were selected in silico using the Homo sapiens reference proteome from UniProt. Non-unique, frequently modified, and peptides containing continuous sequences of R and K (e.g., KR, RK, KK or RR) were excluded when possible. In cases where these were unavoidable, both unmodified and modified forms, as well as peptides with missed cleavages, were analyzed. Details of peptides used are provided in Supplementary Table S1. Synthetic peptides (Thermo Fisher Scientific) and tryptic digests from recombinant proteins were analyzed to determine retention times and fragmentation patterns for the most intense tryptic peptides (2+ or 3+ charge states). These data were used to create a scheduled method with a 4 min retention time window for quantification of peptides in the cell lysate samples. Quantitative analysis utilized a minimum of two peptides per protein, with peptide abundance calculated by summing the integrated peak areas of the top four most intense fragments. Protein abundance was determined by combining peptide-level data. All experiments were performed with three biological replicates.

### Label-free quantitative proteomics

Three biological replicates were analyzed in triplicate, resulting in nine runs per sample group. Peptides were prepared via tryptic digestion as described for targeted mass spectrometry. Desalting of peptides was performed using C18 spin columns (Thermo Fisher Scientific) according to the manufacturer’s instructions. After elution from the C18 columns, peptides were dried and reconstituted in 0.1% FA. LC–MS/MS was conducted on a timsTOF Pro instrument (Bruker Daltonics) coupled to a nanoElute HPLC system (Bruker Daltonics). Peptides were separated on a Bruker15 column (75 µm × 15 cm) with a gradient elution using buffer A (0.1% FA) and buffer B (0.1% FA in acetonitrile), transitioning from 0 to 37% buffer B over 100 min. The timsTOF instrument operated in data-dependent acquisition (DDA) with parallel accumulation serial fragmentation (PASEF) acquiring 10 PASEF scans per cycle with 100 ms accumulation and ramp times. Target intensity was set to 20,000, with dynamic exclusion activated and set to 0.4 min. Quadrupole isolation widths were set to 2 Th for m/z < 700 and 3 Th for m/z > 800.

Data were processed using open workflow^76^ to determine optimal search parameters for MaxQuant^77^ (v2.0.3.0). Alongside default settings, deamidation of asparagine and glutamine were included as dynamic post-translational modifications. The search was conducted against the Human proteome including isoforms downloaded from UniProt (https://www.uniprot.org) in June 2021 and MaxQuant’s built-in contaminants database, using the Andromeda search engine integrated into MaxQuant. The match-between-runs (MBR) feature was enabled to minimize missing values, allowing peptide signals present in one sample but absent in another to be identified by leveraging high mass precision and a restricted retention time window (1 min alignment with a 2 min sliding window). Signals detected through MBR were annotated as ‘By matching’ rather than ‘By MS/MS’ in the output. Both protein and peptide group identifications were filtered to a 1% false discovery rate (FDR). Only unique peptides with high-confidence identifications were included for final protein group assignments. Intensity values for each protein group were normalized using label-free quantification (LFQ) algorithm^78^, requiring at least one unique peptide per protein group.

### Bioinformatic analyses

Log_2_ transformation was applied to the LFQ value to generate log2-LFQ data. The median log2-LFQ value of technical replicates was used to represent each biological replicate, and the median of the three biological replicates was taken as the log2-LFQ value for each protein in the group. Two-sided Student’s *t*-tests^79^ were conducted over log2-LFQ values representing biological replicates using Excel and p-values were calculated. To account for multiple testing, p-values were ranked and adjusted using the Benjamini–Hochberg method^80^, yielding false discovery rate (q-value) estimates. Proteins detected in all control replicates but in none of the treated samples, were assigned an LFQ ratio of − 100 and a q-value of 0.0. Similarly, proteins absent in the controls but present in every treated sample replicate, were assigned an LFQ ratio of 100 and both p- and q-value set to 0.0.

Biological pathway analyses were performed using Ingenuity® Pathway Analysis (IPA) (Ingenuity Systems, www.ingenuity.com)^81^, the PANTHER overrepresentation test (Release 20221013, GO Ontology database, released 2022-07-01)^82,83^ and ShinyGO (http://bioinformatics.sdstate.edu/go/)^84^. Background was set to the entire Supplementary Table S2 and significance threshold at p-value = 0.05 throughout the pathway analyses.

### Dose response assay

Freely cycling cells were harvested with trypsin and resuspended in fresh medium. After counting in a Countess automated cell counter (Thermo Fisher Scientific) 2000 cells/well were seeded in 96 well plates. Medium containing drug was added after 12 and/or 24 h. After drug treatment (for 48 h and/or 60 h), 20 µl 2.5 mM resazurin (Sigma 199303, sterile filtered) per 100 µl culture volume was added and fluorescence measured in a FLUOstar Omega plate reader (BMG Labtech) after 2–4 h of incubation. Background with cell free medium and resazurin was subtracted from every sample.

### CRISPR/Cas9 clones

UNG2 and UNG1+2 gene knockouts of A549 and HeLa cells were made by lentiviral transduction according to the Zang lab protocol^85^, with details and guide RNA DNA oligos as described previously^86^. Vectors expressing no gRNA (Ctrl) and gRNA targeting UNG intron 2 (Int), UNG exon 1A (UNG2 KO), and UNG exon 1B (UNG1+2 KO), were used to generate two independent clones (A, B) of each genotype. Control cells (Ctr) were made with the pGIPZ vector encoding GFP and puromycin resistance but with no guide RNA. Clones were subsequently screened by western blot analysis.

## Supplementary Information

Supplementary Figure S1.

Supplementary Figure S2.

Supplementary Figure S3.

Supplementary Figure S4.

Supplementary Table S1.

Supplementary Table S2.

## Data Availability

Mass spectrometry data can be downloaded from the ProteomeXchange Consortium via the PRIDE partner repository with the dataset identifier PXD051824. (https://www.ebi.ac.uk/pride/archive/projects/PXD051824, Username: reviewer_pxd051824@ebi.ac.uk Password: ITq7Z8ii).

## Abbreviations

AP: Apyrimidinic
BER: Base excision repair
DHFR: Dihydrofolate reductase
DDA: Data dependent acquisition
DSB: Double-strand break
dUTP: Deoxyuridine triphosphate
dUTPase/DUT: Deoxyuridine triphosphate nucleotide hydrolase
ESI: Electrospray ionization
ETC: Electron transport chain
FEN1: Flap endonuclease 1
5-FdU: 5-Fluoro-2-deoxyuridine
5-FU: 5-Fluorouracil
5-FUTP: 5-Fluorouridine triphosphate
FWHM: Full width at half maximum
GART: Trifunctional purine biosynthetic protein adenosine-3
GFP: Green fluorescent protein
Grna: Guide RNA
HAT: Histone acetyltransferase
HDAC: Histone deacetylase
HDACi: Histone deacetylase inhibitor
HRP: Horseradish peroxidase
LFQ: Label-free quantitative
MS275: Entinostat
mtDNA: Mitochondrial DNA
NSCLC: Non-small cell lung cancer
PARP: Poly (ADP-ribose) polymerase
PASEF: Parallel accumulation serial fragmentation
PCNA: Proliferating cell nuclear antigen
PI: Propidium iodide
PMX: Pemetrexed
POLE/D: DNA polymerase epsilon/delta
PRM: Parallel reaction monitoring
RAD51: DNA repair protein RAD51 homolog 1
RER: Ribonucleotide excision repair
RTX: Raltitrexed
SAHA: Vorinostat
TLD: Thymineless death
TOP1: Topoisomerase 1
TYMS: Thymidylate synthase
UNG2: Uracil-DNA glycosylase isoform 2 (nuclear)
UTP: Uridine-5-triphosphate
