# Supplementary material for: Cytotoxic mechanisms of pemetrexed and HDAC inhibition in non-small cell lung cancer cells involving ribonucleotides in DNA
Source: Sci Rep. 2025 Jan 15;15:2082. doi: 10.1038/s41598-025-86007-w (PMC11736037; doi:10.1038/s41598-025-86007-w)
Supplement: Supplementary file 1 — Supplementary Figure S1. [file 41598_2025_86007_MOESM1_ESM.pdf]

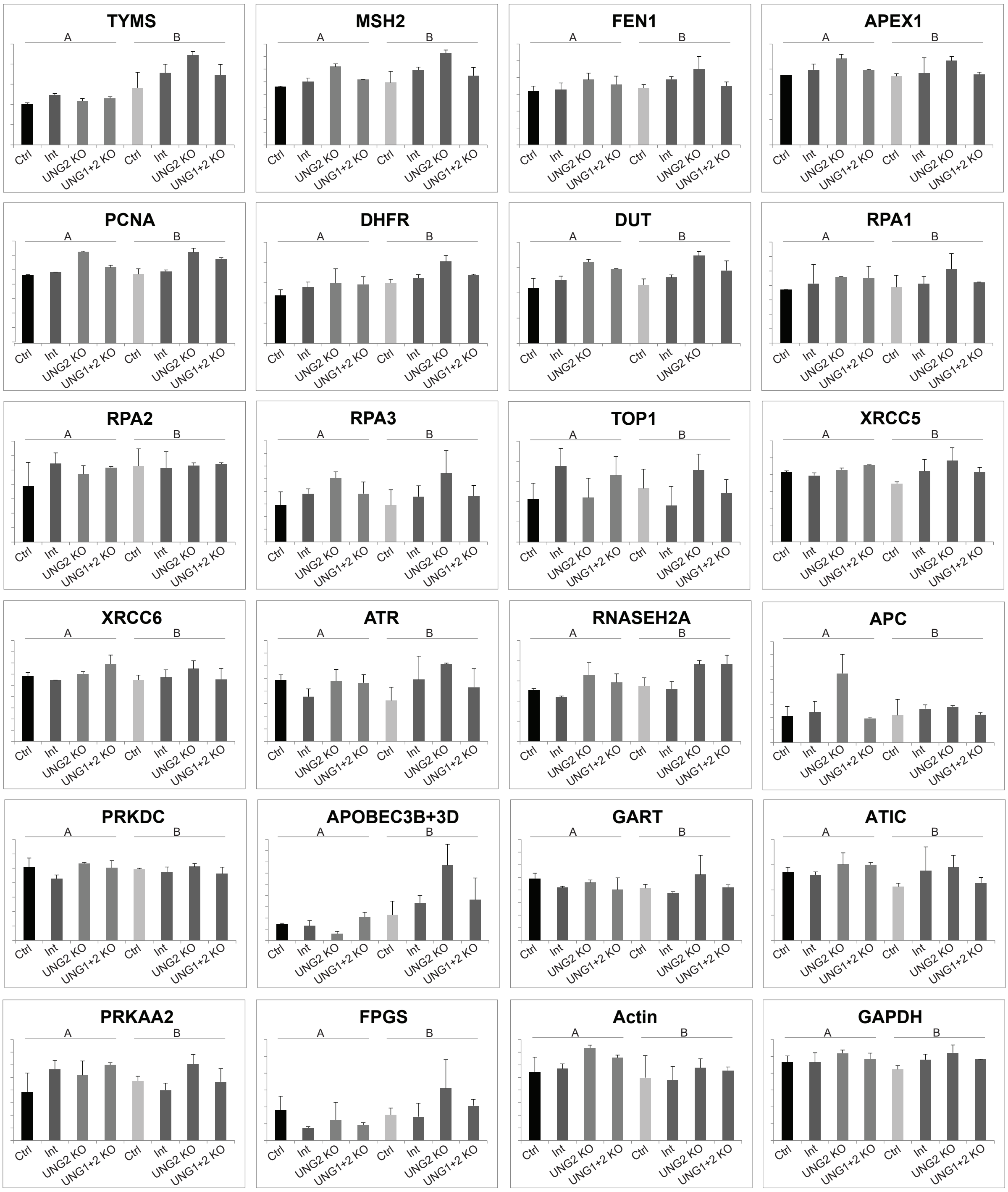

Supplementary Figure S1. A549 UNG2 KO, UNG1+2 KO and control cells transduced with virus without gRNA (Ctrl) or rRNA targeting Intron 2 in UNG (Int). A and B clones of each (indicated above bars) were subjected to PRM targeted MS analysis of selected proteins involved in pyrimidine metabolism and DNA repair to identify potentially relevant off-target effects. Y axes represent relative protein abundancies and error bars represent the standard deviations. No significant off-target effects were detected.
