# Supplementary material for: Cytotoxic mechanisms of pemetrexed and HDAC inhibition in non-small cell lung cancer cells involving ribonucleotides in DNA
Source: Sci Rep. 2025 Jan 15;15:2082. doi: 10.1038/s41598-025-86007-w (PMC11736037; doi:10.1038/s41598-025-86007-w)
Supplement: Supplementary file 2 — Supplementary Figure S2. [file 41598_2025_86007_MOESM2_ESM.pdf]

Full blots related to Figure 1D

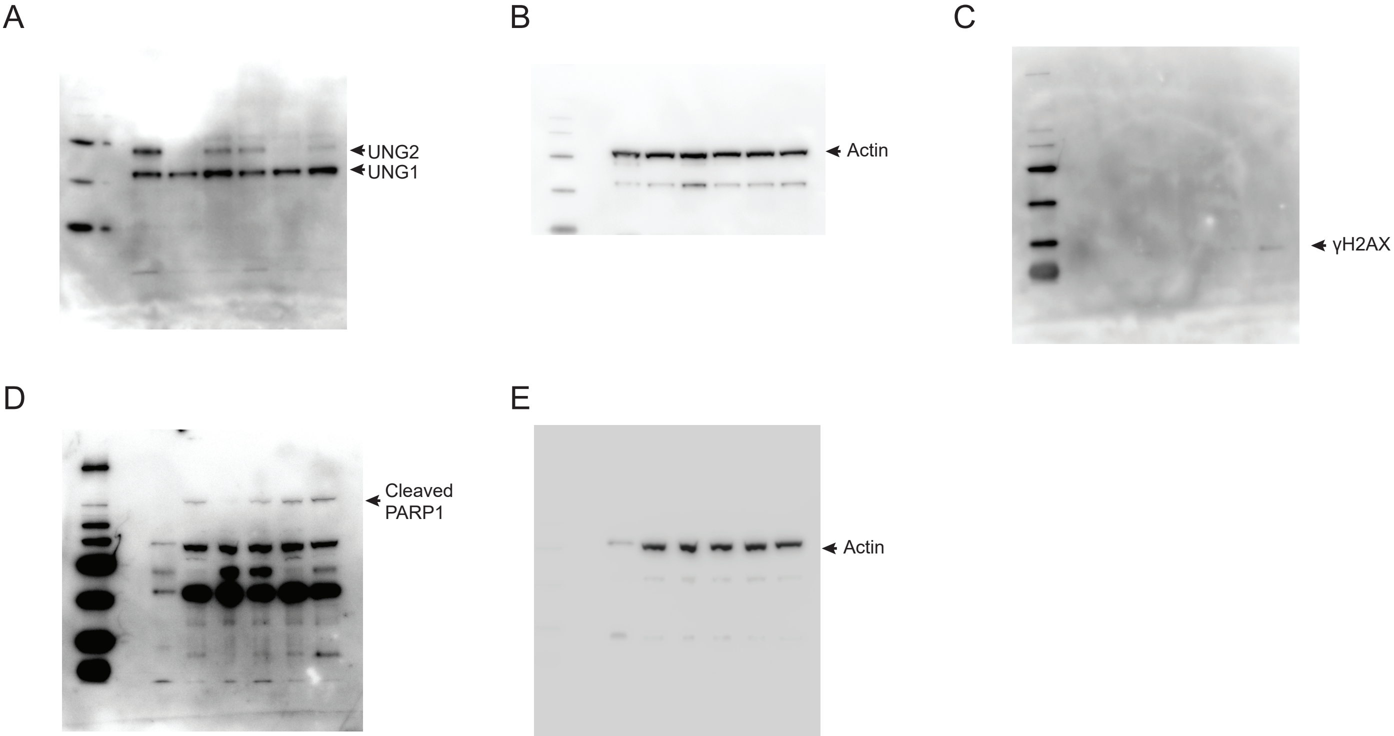

Full blots related to Figure 2D

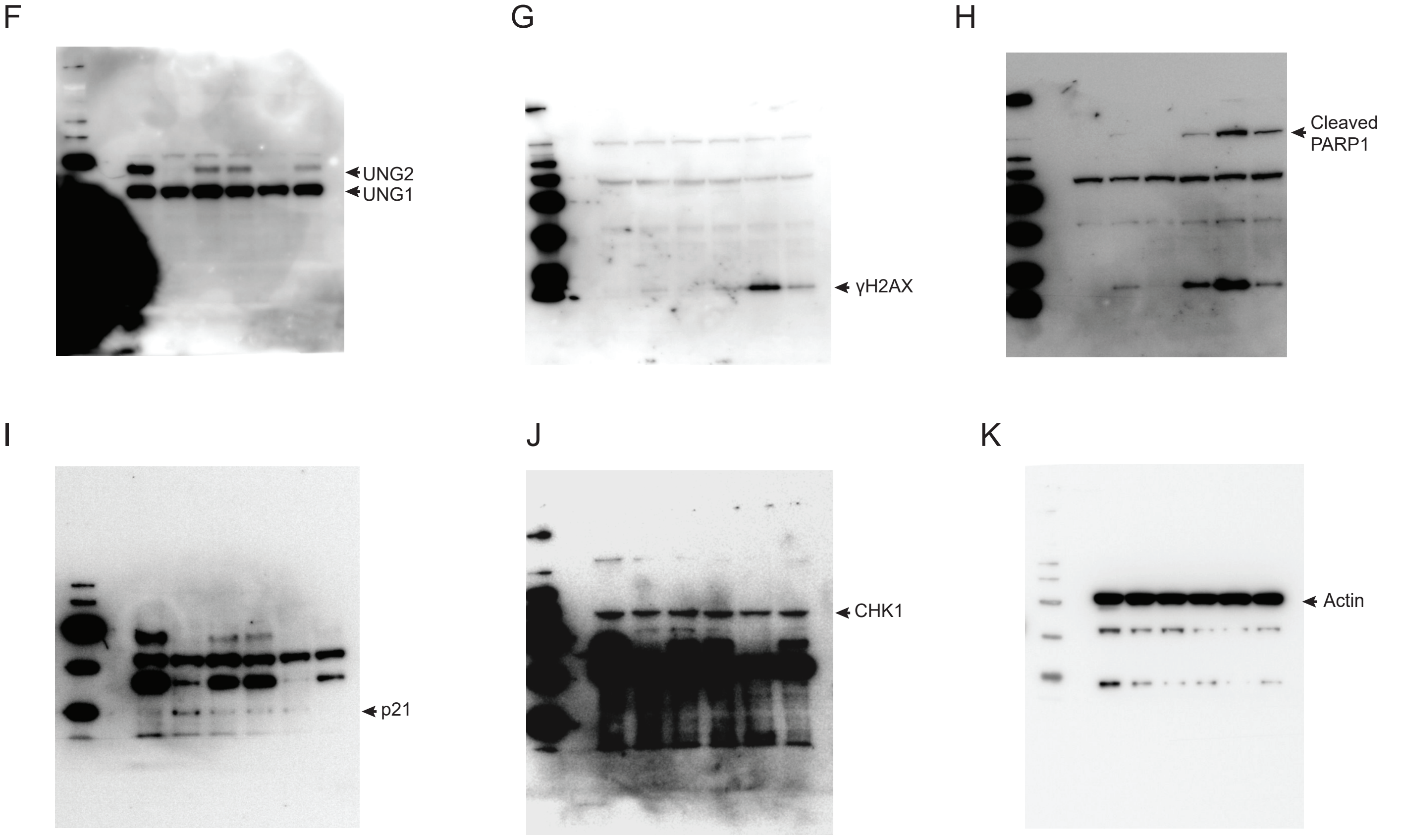

Supplementary Figure S2. A) Full blot of Figure 1D, UNG1 and UNG2, **B)** Stripped and reprobed A with Actin Ab, **C)** Full blot of Figure 1D, γH2AX **D)** Stripped and reprobed C with cleaved PARP1 Ab, **E)** Stripped and reprobed D with Actin Ab, **F)** Full blot of Figure 2D, UNG1 and UNG2, **G)** Stripped and reprobed with γH2AX Ab **H)** Stripped and reprobed G with cleaved PARP1 Ab, **I)** stripped and reprobed H with p21 Ab, **J)** Stripped and reprobed I with CHK1 Ab, **K)** Stripped and reprobed J with Actin Ab.
