# Supplementary material for: Cytotoxic mechanisms of pemetrexed and HDAC inhibition in non-small cell lung cancer cells involving ribonucleotides in DNA
Source: Sci Rep. 2025 Jan 15;15:2082. doi: 10.1038/s41598-025-86007-w (PMC11736037; doi:10.1038/s41598-025-86007-w)
Supplement: Supplementary file 3 — Supplementary Figure S3. [file 41598_2025_86007_MOESM3_ESM.pdf]

Full blots related to Figure 2E

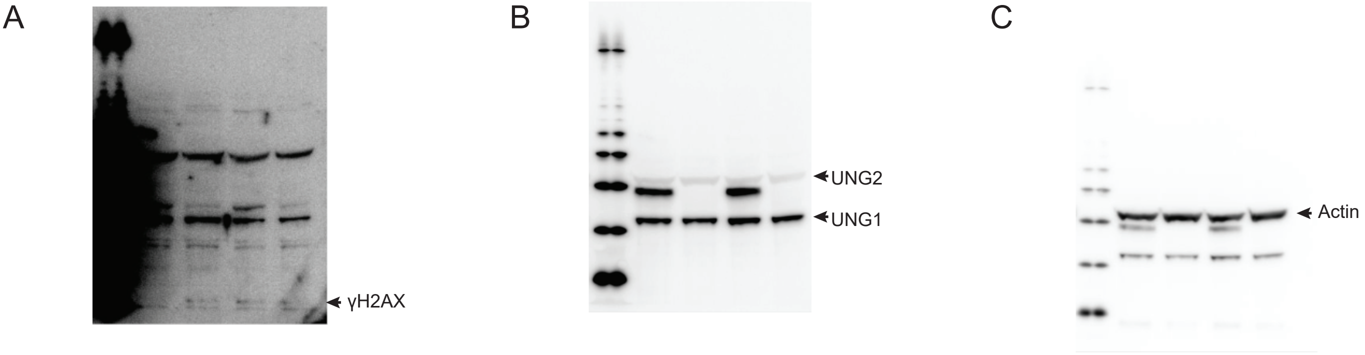

Full blots related to Figure 2F

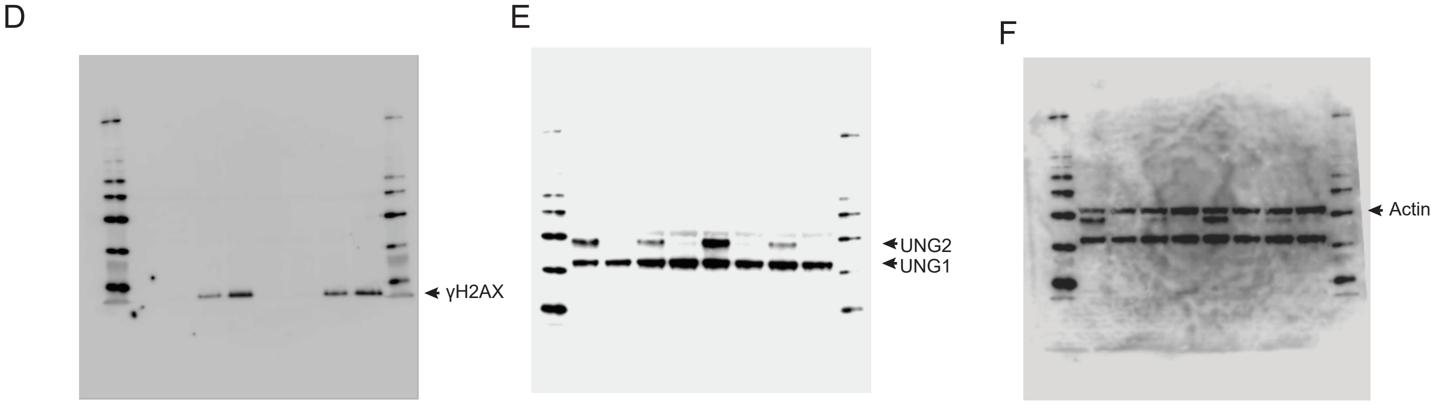

Full blots related to Figure 2G

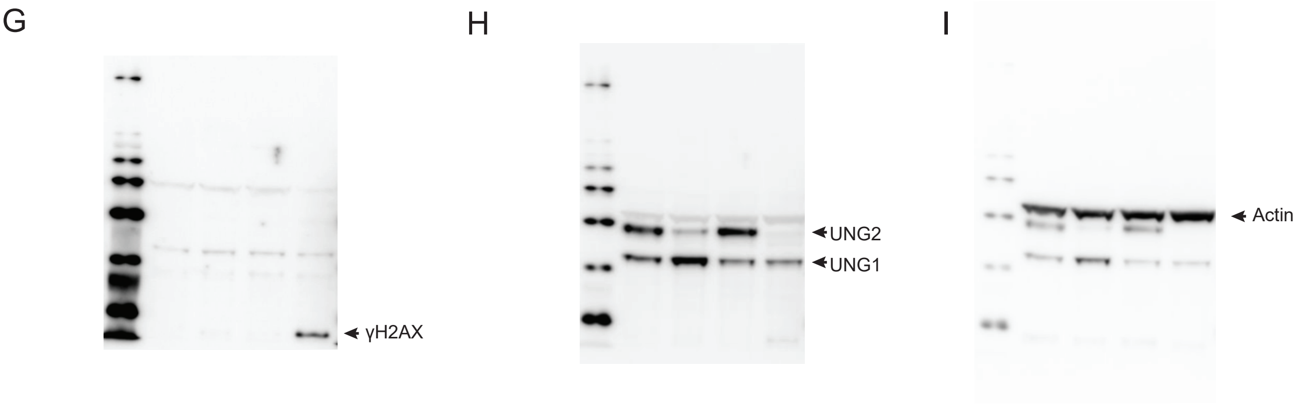

Supplementary Figure S3. A) Full blot of Figure 2E, γH2AX, **B)** Stripped and reprobed A with UNG1/2 Ab **C)** Stripped and reprobed B with Actin Ab, **D)** Full blot of Figure 2F, γH2AX, **E)** Stripped and reprobed D with UNG1/2 Ab **F)** Stripped and reprobed E with Actin Ab, **G)** Full blot of Figure 2G, γH2AX, **H)** Stripped and reprobed G with UNG1/2 Ab, **I)** Stripped and reprobed D with Actin Ab,
