# Supplementary material for: Cytotoxic mechanisms of pemetrexed and HDAC inhibition in non-small cell lung cancer cells involving ribonucleotides in DNA
Source: Sci Rep. 2025 Jan 15;15:2082. doi: 10.1038/s41598-025-86007-w (PMC11736037; doi:10.1038/s41598-025-86007-w)
Supplement: Supplementary file 4 — Supplementary Figure S4. [file 41598_2025_86007_MOESM4_ESM.pdf]

Full blots related to Figure 3A

A

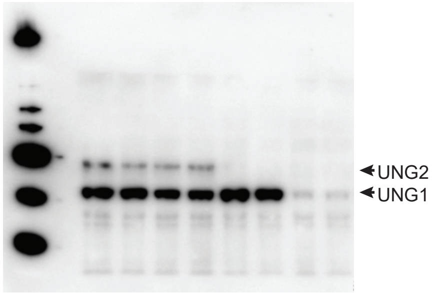

B

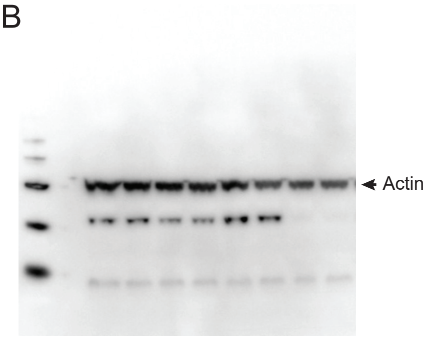

Full blots related to Figure 3D, upper panel

C

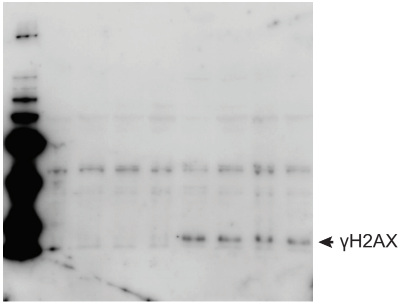

D

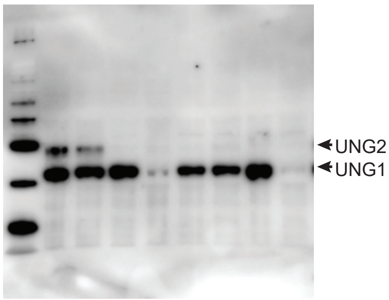

E

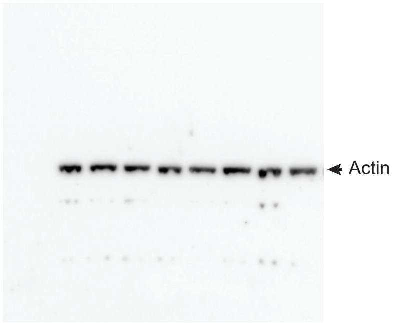

Full blots related to Figure 3D, bottom panel

F

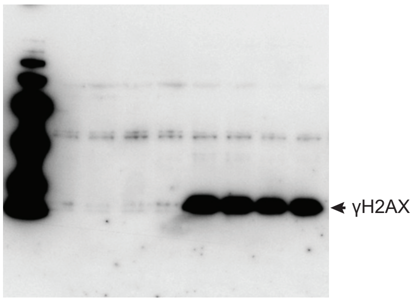

G

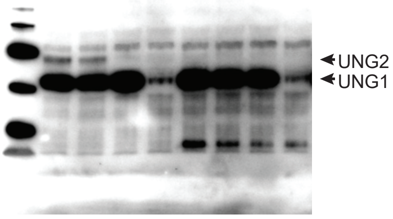

H

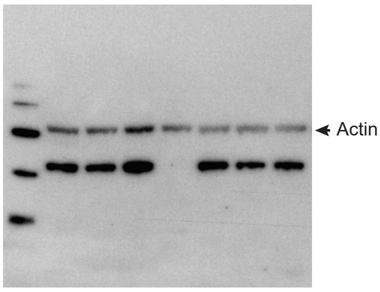

Supplementary Figure S4. A) Full blot of Figure 3A, UNG1 and UNG2, **B)** Stripped and reprobed A with Actin Ab, **C)** Full blot of Figure 3D, upper panel, γH2AX **D)** Stripped and reprobed C with UNG1/2 Ab, **E)** Stripped and reprobed D with Actin Ab, **F)** Full blot of Figure D, lower panel, γH2AX, **G)** Stripped and reprobed with UNG1/2 Ab **H)** Stripped and reprobed G with Actin Ab.
